# Gelatin-containing porous polycaprolactone PolyHIPEs as substrates for 3D breast cancer cell culture and vascular infiltration

**DOI:** 10.3389/fbioe.2023.1321197

**Published:** 2024-01-08

**Authors:** Caitlin E. Jackson, Iona Doyle, Hamood Khan, Samuel F. Williams, Betül Aldemir Dikici, Edgar Barajas Ledesma, Helen E. Bryant, William R. English, Nicola H. Green, Frederik Claeyssens

**Affiliations:** ^1^ The Kroto Research Institute, Materials Science and Engineering, University of Sheffield, Sheffield, United Kingdom; ^2^ Insigneo Institute for in Silico Medicine, The Pam Liversidge Building, University of Sheffield, Sheffield, United Kingdom; ^3^ Department of Infection, Immunity and Cardiovascular Disease, Royal Hallamshire Hospital, The University of Sheffield, Sheffield, United Kingdom; ^4^ Department of Bioengineering, Izmir Institute of Technology, Urla, Türkiye; ^5^ Department of Chemistry, The University of Sheffield, Sheffield, United Kingdom; ^6^ School of Medicine and Population Health, University of Sheffield, Sheffield, United Kingdom; ^7^ Norwich Medical School, University of East Anglia, Norwich, United Kingdom

**Keywords:** gelatin, polyHIPE, CAM assay, PCL (polycaprolactone), vascularisation, angiogenesis

## Abstract

Tumour survival and growth are reliant on angiogenesis, the formation of new blood vessels, to facilitate nutrient and waste exchange and, importantly, provide a route for metastasis from a primary to a secondary site. Whilst current models can ensure the transport and exchange of nutrients and waste via diffusion over distances greater than 200 μm, many lack sufficient vasculature capable of recapitulating the tumour microenvironment and, thus, metastasis. In this study, we utilise gelatin-containing polymerised high internal phase emulsion (polyHIPE) templated polycaprolactone-methacrylate (PCL-M) scaffolds to fabricate a composite material to support the 3D culture of MDA-MB-231 breast cancer cells and vascular ingrowth. Firstly, we investigated the effect of gelatin within the scaffolds on the mechanical and chemical properties using compression testing and FTIR spectroscopy, respectively. Initial *in vitro* assessment of cell metabolic activity and vascular endothelial growth factor expression demonstrated that gelatin-containing PCL-M polyHIPEs are capable of supporting 3D breast cancer cell growth. We then utilised the chick chorioallantoic membrane (CAM) assay to assess the angiogenic potential of cell-seeded gelatin-containing PCL-M polyHIPEs, and vascular ingrowth within cell-seeded, surfactant and gelatin-containing scaffolds was investigated via histological staining. Overall, our study proposes a promising composite material to fabricate a substrate to support the 3D culture of cancer cells and vascular ingrowth.

## 1 Introduction

Angiogenesis is the process through which new vasculature is formed from an existing network and is a key process to ensure cell survival and maintenance, facilitating oxygen and nutrient delivery, and waste removal (Adair and Montani, 2010; Rouwkema and Khademhosseini, 2016). Moreover, angiogenesis is critical for tumour survival, maintenance and growth, as well as providing a route for cancer cell metastasis (Nishida et al., 2006; Lugano et al., 2020). However, many current *in vitro* models lack sufficient vasculature to fully recapitulate tumour-driven angiogenesis, tumour growth and metastasis. Therefore, there is a need to design improved *in vitro* culture substrates to support tumour cell culture and growth whilst additionally facilitating tumour-driven angiogenesis and vascular ingrowth.

Substrates for *in vitro* culture are commonly fabricated using polymers, either natural or synthetic (Langer and Tirrell, 2004; Kohane and Langer, 2008; Place et al., 2009). Whilst natural polymers, such as collagen and Matrigel, better recapitulate the architecture of the microenvironment (Habanjar et al., 2021) and lend themselves to optical microscopy analysis techniques better than synthetic polymers, they are often fabricated as hydrogels, and as such, the resultant mechanical properties of the gels can cause challenges in scaffold handling. Synthetic polymers can be fabricated consistently, at low cost, are easier to produce and can often be chemically or mechanically tuned, producing scaffolds which can be easily handled (Rijal et al., 2017; Donnaloja et al., 2020; Reddy et al., 2021). Thus, a substrate fabricated with a combination of natural and synthetic polymers could provide a better solution for improved *in vitro* cell culture, resulting in a substrate which has architecture recognisable by cells whilst still easily handled.

We have previously reported on the use of gelatin to both chemically and mechanically tune porous polymer substrates (Furmidge et al., 2023). Gelatin is a biodegradable and biocompatible polymer with low toxicity that is a molecular derivative of type I collagen, therefore, it has the capability to biologically perform similarly to collagen and is a suitable substitute (Bello et al., 2020). Furthermore, gelatin is readily available, can be extracted from multiple sources and is more cost-effective than extracellular matrix (ECM) proteins such as collagen, laminin and fibronectin (Bello et al., 2020; Lukin et al., 2022).

Due to the amphiphilic properties of gelatin, it has been previously utilised as a surfactant, albeit a weak surfactant and is capable of stabilising emulsions (Aldemir Dikici and Claeyssens, 2020; Zhang et al., 2020; Furmidge et al., 2023) via lowering the interfacial energy of the oil-water interface.

Our previous study demonstrated how the inclusion of gelatin within the internal phase of PCL polyHIPEs led to a significant increase in pore size of the resulting scaffold (Table 1) (Furmidge et al., 2023). Thus, we hypothesised that such increases in the pore size could enable increased vessel ingrowth. Therefore, this study investigates the use of gelatin-containing polyHIPEs as a substrate to support 3D breast cancer cell growth and facilitate angiogenesis. We initially assessed the impact of gelatin within the scaffold on mechanical properties and the cancer cell metabolic activity before using the pre-existing vascular network from an *ex ovo* chick chorioallantoic membrane (CAM) assay to assess the vascular ingrowth of the CAM vessels. Furthermore, we combined 3D cancer cell culture on the substrates as a tumour tissue mimic within the CAM assay, assessing the validity of our approach using a porous polymer substrate to recapitulate angiogenesis surrounding tumour tissue.

**TABLE 1:** The pore size (Furmidge et al., 2023) and stiffness of PCL-M polyHIPEs fabricated with different combinations of 10% surfactant and 5% gelatin (mean ± SD).

PCL-M PolyHIPE	Pore size (µm) (Furmidge et al., 2023)	Stiffness (MPa)
G0S10	53 ± 19	0.91 ± 0.23
G5S10	39 ± 24	2.68 ± 0.60
G5S0	80 ± 43	1.52 ± 0.20

## 2 Materials

Photoinitiator (2,4,6-Trimethylbenzoyl Phosphine Oxide/2-Hydroxy-2- Methylpropiophenone blend), Dulbecco’s modified Eagle media (DMEM), fetal bovine serum (FBS), pencillin/streptomycin (PS), L-glutamine, trypsin, formaldehyde, resazurin sodium salt, type A gelatine from porcine skin, isopentane and haematoxylin solution were purchased from Sigma Aldrich. Chloroform, toluene, ethanol, acetone and methanol were purchased from Fisher Scientific. The surfactant, Hypermer B246 was received as a sample from Croda (Goole, United Kingdom). The optimal cutting temperature-tissue freezing medium (OCT-TFM) was purchased from CellPath, the VectaMount aqueous mounting medium was purchased from Vector and the eosin solution was purchased from Acros Organics. High molecular weight 4-arm methacrylated polycaprolactone (PCL-M, 20,331 g/mol, 95% methacrylated) was synthesised in the laboratory [a general synthesis method is given in Aldemir Dikici et al. (2019)].

## 3 Methods

### 3.1 PCL-M PolyHIPE fabrication

0.4 g PCL-M and 10 wt% surfactant were heated to melt the surfactant and PCL-M. 0.6 g of 60 wt% chloroform and 40 wt% toluene solvent mixture and 0.03 g photoinitiator were added to the PCL-M-surfactant mixture respectively. The contents were mixed (250 rpm) using a magnetic stirrer (20 mm × 7 mm) for 3 min at 37°C. Once homogeneous, 2 mL of the internal phase (water or 5% gelatin solution prepared with water (wt/v)) was added dropwise and the emulsion was mixed for 5 min. Three compositions were prepared: i) 10 wt% surfactant with water as an internal phase (G0S10), ii) 10 wt% surfactant with 5% gelatin solution as an internal phase (G5S10) and iii) 0 wt% surfactant with 5% gelatin solution as an internal phase (G5S0).

### 3.2 Polymerisation of PCL-M HIPEs

Emulsions were polymerised in a transparent 2 mL syringe. All samples were cured using ultraviolet (UV) light for 5 min on both sides using the OmniCure Series 1,000 system (100 W, Lumen Dynamics, Canada), with 18 W/cm^2^ reported light density and spectral output from 250–600 nm. The resulting polyHIPEs were removed from the syringe and washed in 100% ethanol for 24 h before washing in 70% ethanol for 48 h, changing the ethanol after each 24 h period. Following this, ethanol was gradually replaced with deionised water for 3 days, changing the water after each 24 h period. All polyHIPE samples were washed and stored in dH_2_0 at room temperature.

### 3.3 Mechanical characterisation

The compressive modulus of the PCL-M polyHIPEs was calculated by compressive mechanical testing (MultiTest 2.5–dv, Mecmesin, Slinford, United Kingdom), using the 250 N load cell at room temperature. Samples were cut into approximately 1 cm cylinders using a scalpel and placed between two compression plates. The compressive tests were performed on each sample at a rate of 1 N/s until the maximum load of 250 N was reached. The stiffness was calculated from the gradient of the initial linear region of the stress-strain curve for each sample.

### 3.4 Chemical characterisation using fourier transform infrared spectroscopy (FTIR)

Measurements were collected using an Agilent 4300 spectrometer fitted with a diamond 3-Bounce-2-Pass attenuated total reflection (ATR) crystal and a mercury cadmium telluride detector (Agilent Technologies, Santa Clara CA, United Kingdom). Data between 4000 cm^−1^ and 1,000 cm^−1^ was obtained by collecting 32 scans at 4 cm^−1^ resolution. All spectra were normalised to the PCL peak (1722 cm^-1^). Spectral processing was conducted using Spectragryph (v1.2.15, 2020).

### 3.5 General cell culture

MDA-MB-231 cells (Aldrich, 2023) were used to evaluate the proliferation of cancer cells within gelatin-containing PCL-M polyHIPEs. The MDA-MB-231 cells were purchased from Merck (ECACC) and transduced to express luciferase2 and mStrawberry by transfection with a transposon and the transposase PiggyBac using methodology developed previously (English et al., 2017). The cells were transduced and selected with puromycin and stocks frozen within 5 passages and then used within 30 passages of receipt from ECACC. The cells were thawed, transferred to media (DMEM supplemented with 10% FBS, 1% PS, 1% L-glutamine) and centrifuged at 95 g for 5 min. The cell pellet was resuspended in fresh media with 1 μg/mL puromycin and cultured until 90% confluence with media changes every 3 days. Puromycin was removed from the media 24 h before each experiment.

### 3.6 MDA-MB-231 cell seeding on PCL-M polyHIPE scaffolds

To initially characterise cell-scaffold interactions, polyHIPE discs (8 mm diameter and 1 mm depth) were used. To sterilise, all scaffolds were washed in ethanol followed by dH_2_O. Once reaching 90% confluency, cells were detached from the cell culture flask using trypsin. After 4 min the trypsin was neutralised with cell culture media (ratio of 1:2 respectively), followed by centrifugation (95 × g for 5 min) and resuspended in fresh media before counting using the trypan blue exclusion method to assess cell viability. For cell viability and CAM assays, 25 μL of MDA-MB-231 cells at 2 × 10^6^ cells/mL were transferred onto each scaffold and left for 30 min in the incubator (37°C and 5% CO_2_) to allow for cell attachment. After 30 min, fresh media was placed in each well and incubated for 7 days with fresh media replaced every 2–3 days.

### 3.7 Cell viability on PCL-M polyHIPE scaffolds

The viability of cells on the scaffold was assessed using the resazurin reduction (RR) assay. 1 mM resazurin stock solution was diluted in cell culture media to form a 10% v/v resazurin working solution. The media was removed and discarded from each well and a further 0.5 mL of the working solution was added to each well. The well plate was protected from light and incubated for 4 h at 37°C. An orbital rocker (30 rpm) was used in the incubator to ensure full penetration of the resazurin working solution. 150 μL was taken, in triplicate, from each scaffold and transferred to a 96 well plate. A fluorescence microplate reader (BioTek FLx800, Agilent BioTek, Santa Clara, CA, United States) was used to read the fluorescence of each well at an excitation wavelength of 540 nm and an emission wavelength of 630 nm. The working solution was removed from the scaffolds, and each scaffold was further washed with PBS three times before adding fresh cell culture media and continuing incubation. The assay was performed at days 1, 3 and 7.

### 3.8 VEGF ELISA

The concentration of VEGF in the supernatant of cell-seeded scaffolds was determined using the Human VEGF ELISA kit, according to the manufacturer’s instructions (Abcam, United Kingdom). The optical density was measured on an absorbance microplate reader (BioTek ELx800, Agilent BioTek, Santa Clara, CA, United States) set to 450 nm.

### 3.9 CAM assay

The *ex ovo* CAM assay, as described by Mangir et al. (2019) was used to study the vascularisation of gelatin-containing, cell-seeded polyHIPEs. Briefly, pathogen-free fertilised eggs (*Gallus domesticus*), obtained from Med Eggs (Fakenham, United Kingdom), were cleaned with 20% v/v industrial methylated spirits (IMS) before incubating in humidified (45%) hatching incubators (Rcom King Suro Max-20, P&T Poultry, Powys, Wales) at 38°C and for 3 days. After 3 days the eggs were cracked into sterile 100 mL weigh boats with 3 mL of PBS + 1% v/v penicillin–streptomycin solution (100 IU/mL–100 mg/mL) (Supplementary Figure S1). The eggs were further incubated at 38°C in a cell culture incubator (Binder, Tuttlingen, Germany). At day 7 of embryonic development, 500 µm polyHIPE discs sectioned using a vibratome (5100 mz, Campden Instruments, Loughborough, United Kingdom), seeded as described in Section 3.6, were implanted within the boundaries of the CAM and incubated for a further 6 days. The scaffolds were placed with the non-seeded surface in contact with the CAM to create a chemotactic gradient through the polyHIPE scaffold from the CAM surface. At day 13 of embryonic development, the CAM was imaged using a digital camera and MicroCapture software (version 2.0). Body lotion (Extracts, Tesco, Welwyn Garden City, UK) was injected into the surrounding area of the sample to provide contrast between blood vessels and the sample (Supplementary Figure S2). Following imaging, all embryos were sacrificed by the end of day 13 of embryonic development. Within each condition, initially 8 scaffolds were placed within the boundary of the CAM of individual eggs. This allowed for compensation when the foetus became unviable or for when the scaffold was not in an optimal position for imaging and analysis following the growth of the foetus. For each condition, the 3 scaffolds which were most optimally positioned were used for image analysis and data collection.

### 3.10 Morphometric quantification of the angiogenesis

Three images from each group were quantified using IKOSA software (CAM Assay, KML Vision GmbH) to assess the total vessel length and area and the number of branching points. Furthermore, a modified version of a well-established method (Barnhill and Ryan, 1983; Eke et al., 2017; Mangir et al., 2019; Dikici et al., 2020; Dikici et al., 2021) was used to assess the number of vessels. Briefly, the following parameters were set to all images in Adobe Photoshop (PS) to improve the ability to discern between the blood vessels; brightness and contrast; −50/10, unsharp; 50/10/0, smart sharpen; 100/5 with Gaussian blur, reduced noise; 5/0/0/50, contrast; 100, and contrast; 20-100. A new layer was created in PS, and all discernible vessels touching the scaffolds were drawn digitally using a Wacom Intuos Pro Medium Tablet with a 2 pixels size-hard round brush. The number of blood vessels was calculated by counting the total count of the vessels touching the border of the scaffolds (Aldemir Dikici et al., 2020).

### 3.11 Haematoxylin and Eosin (H&E) staining

On day 13, following the CAM assay the polyHIPE scaffolds and a surrounding area of tissue were excised from the membrane and were fixed with 3.7% w/v formaldehyde for 30 min at room temperature. The samples were further washed with PBS and stored in 70% ethanol. The excess CAM tissue was trimmed from the edges of the scaffold, and the scaffold was sectioned into 2 semi-circular sections before placing the sections in the cryo-mould with OTC-TFM and freezing in isopentane. The cryo-moulds were placed in liquid nitrogen for 7 min before sectioning on a cryostat (CM 1900, Leica, Germany), 16 µm slices were mounted onto the surface of Thermo SuperFrost^®^ Plus slides. For H&E staining, samples were air dried for 2 h before freezing in a 50% v/v acetone and methanol mixture for 15 min. The slides were washed in PBS for 1 min followed by a wash in H_2_O for 3 min. Slides were stained in haematoxylin for 15 s, rinsed for 10 min in H_2_O and dehydrated in 70% and 90% ethanol for 1 min each. Slides were further stained in eosin for 8 s before washing in 100%, 95% and 70% ethanol for 1 min each and mounted using aquamount medium.

### 3.12 Statistical analysis

Statistical analysis was carried out using the analysis software GraphPad Prism (Version 9.4.1, CA, United States). All data was analysed using one-way or two-way analysis of variance (ANOVA) followed by Dunnett T3 (*n* < 50) multiple comparisons test. Error bars on graphs indicate standard deviation, and all *n* values are given in figure captions where relevant. Statistical significance on graphs is represented as *p*-value < 0.033 (*), 0.002 (**) and 0.001 (***).

## 4 Results

### 4.1 Mechanical characterisation

**FIGURE 1:**
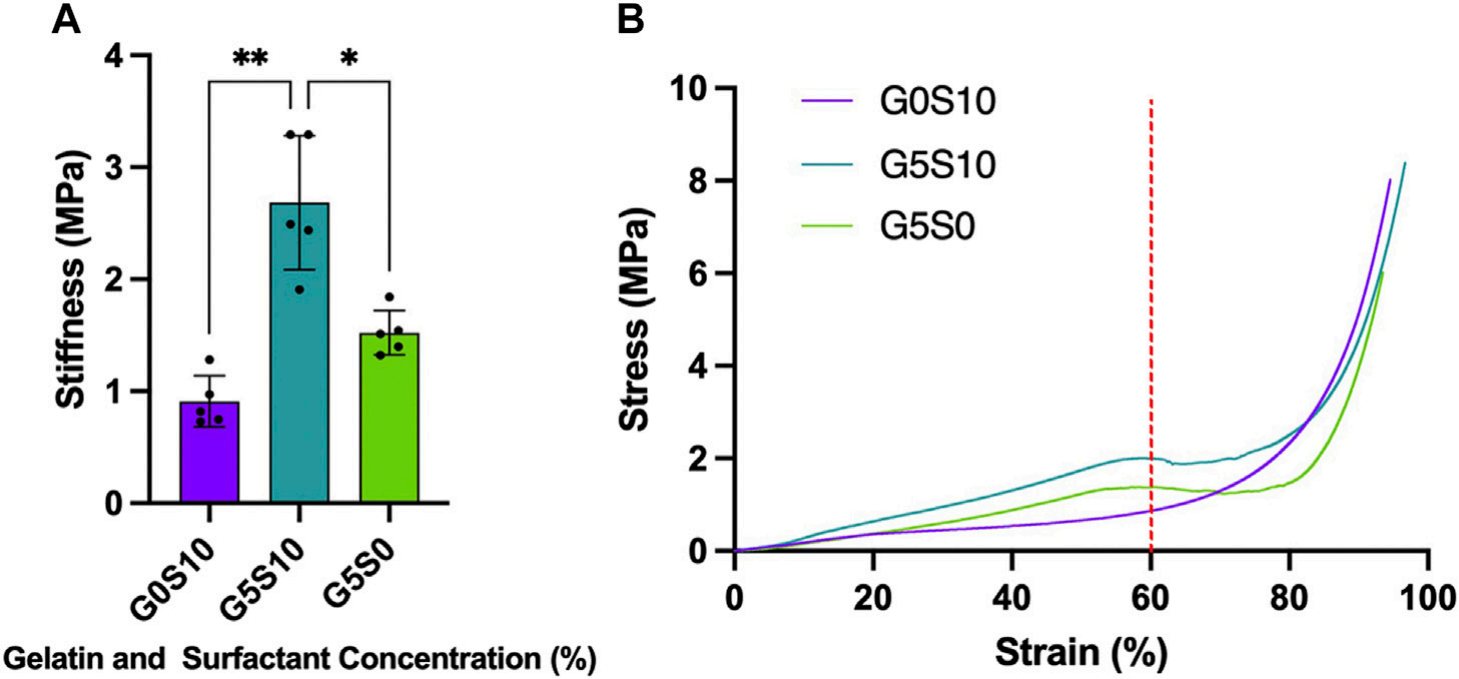
Stiffness of PCL-M polyHIPEs fabricated with different combinations of 10% surfactant and 5% gelatin displaying **(A)** the mean stiffness ± SD (*n* = 5, * *p* < 0.033, ** *p* < 0.002) and **(B)** representative stress-strain curves of each PCL-M polyHIPE condition (red dotted line indicates 60% strain).

The compression tests were conducted under wet conditions, in which the scaffolds had been washed and pre-soaked in deionised water before the experiment. The inclusion of gelatin within the internal phase of the polyHIPE resulted in a significant increase in stiffness independent of whether the scaffold was fabricated with additional surfactant (Table 1; Figure 1A). The stress-strain curves of the gelatin-containing scaffolds demonstrated a deviation in the curve at ∼60% strain, resulting in an S-shaped curve compared to the smooth J-shaped curve observed in the control surfactant-only polyHIPE (G0S10) (Figure 1B).

### 4.2 Biological characterisation

**FIGURE 2:**
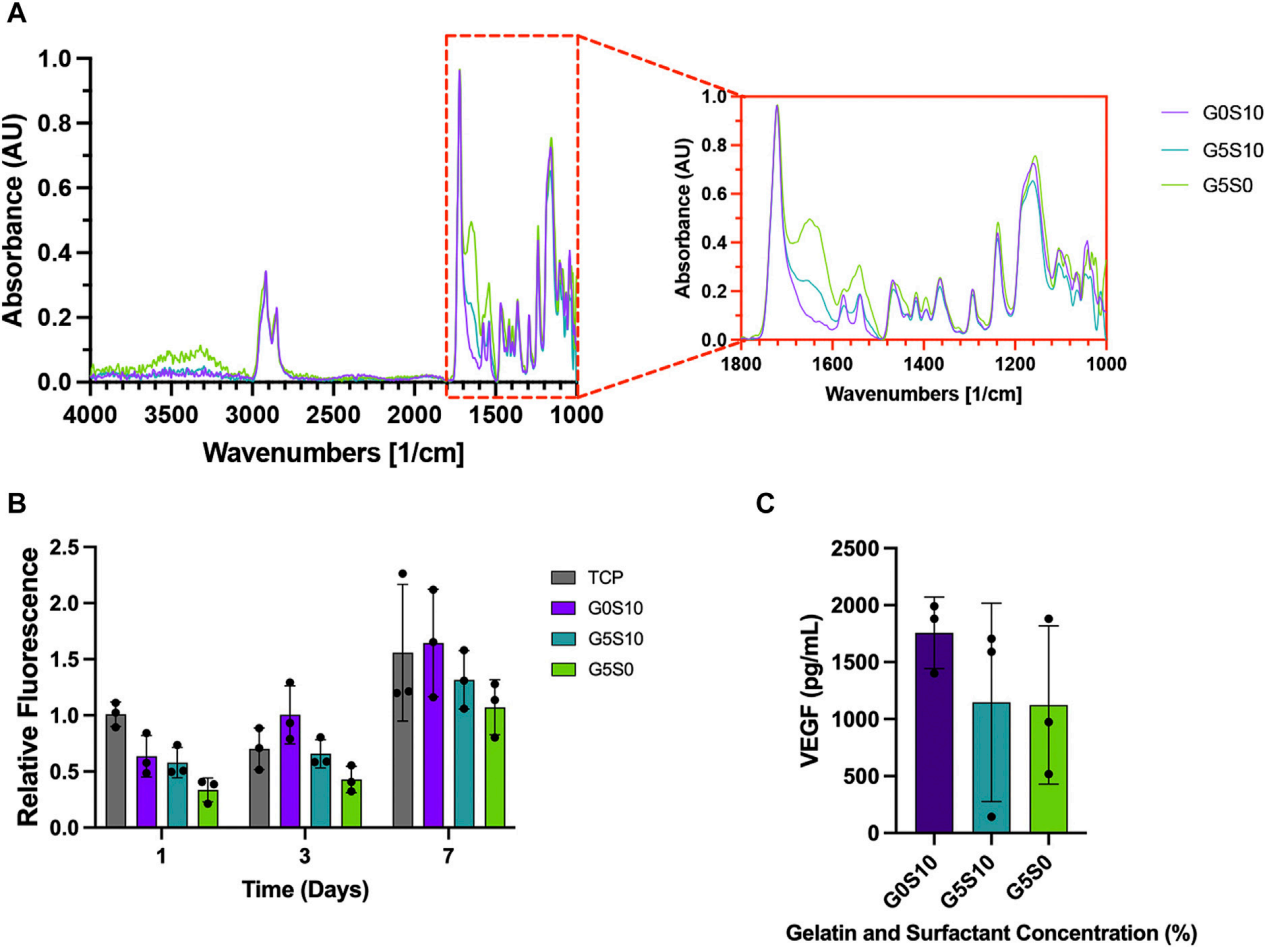
Chemical and biological assessment of PCL–M polyHIPEs containing gelatin. **(A)** The mid-infrared spectrum of PCL-M polyHIPEs containing gelatin, the red callout indicates the fingerprint region. **(B)** The metabolic activity of MDA-MB-231 cells via a resazurin reduction assay across 7 days (mean ± SD, *N* = 3, *n =* 3). **(C)** The concentration of VEGF expressed by MDA-MB-231 cells following 7 days of culture on PCL-M polyHIPEs containing gelatin (mean ± SD, *N* = 3, *n* = 2).

Attenuated total reflection Fourier transform infrared (ATR-FTIR) spectroscopy was used to verify the presence of gelatin in the scaffolds post-processing and before cell seeding. The spectra of all PCL-M polyHIPE samples show characteristic bands for 4 arm PCL, observed at 2,940 cm^−1^ and 2,860 cm^−1^ (Figure 2A), caused by asymmetric and symmetric CH_2_ stretching, respectively. The fingerprint region also shows strong absorption peaks attributed to PCL at 1722 cm^−1^ (ester carbonyl stretching), 1,290 cm^−1^ (C-O and C-C stretching), 1,240 cm^-1^, (asymmetric C-O-C stretching) and 1,170 cm^-1^ (symmetric C-O-C stretching). Furthermore, the fingerprint region of the spectra of the gelatin-containing polyHIPEs (G5S10 and G5S0) also include peaks at 1,630 cm^−1^ (C=O stretching vibration), corresponding to the amide I band. The intensity of the amide I bands is higher and more prominent in the spectrum of the gelatin-only PCL-M polyHIPE.

A 7-day resazurin reduction assay was utilised to assess the metabolic activity of MDA-MB-231 cells on PCL-M scaffolds containing gelatin. There was a significant increase in metabolic activity across the 7-day period for all PCL-M polyHIPE scaffolds (Figure 2B). Furthermore, at each time point, there was no significant difference between the PCL-M polyHIPE scaffolds and the control condition (tissue culture plastic, TCP). A VEGF ELISA was used to quantify the concentration of VEGF expressed by MDA-MB-231 cells following 7 days of culture on PCL-M polyHIPEs. There was no significant difference in the expression of VEGF between the different PCL-M polyHIPEs containing gelatin ± surfactant and standard surfactant-only PCL-M polyHIPEs (Figure 2C).

### 4.3 Ex ovo chorioallantoic membrane assay to assess angiogenic potential

**FIGURE 3:**
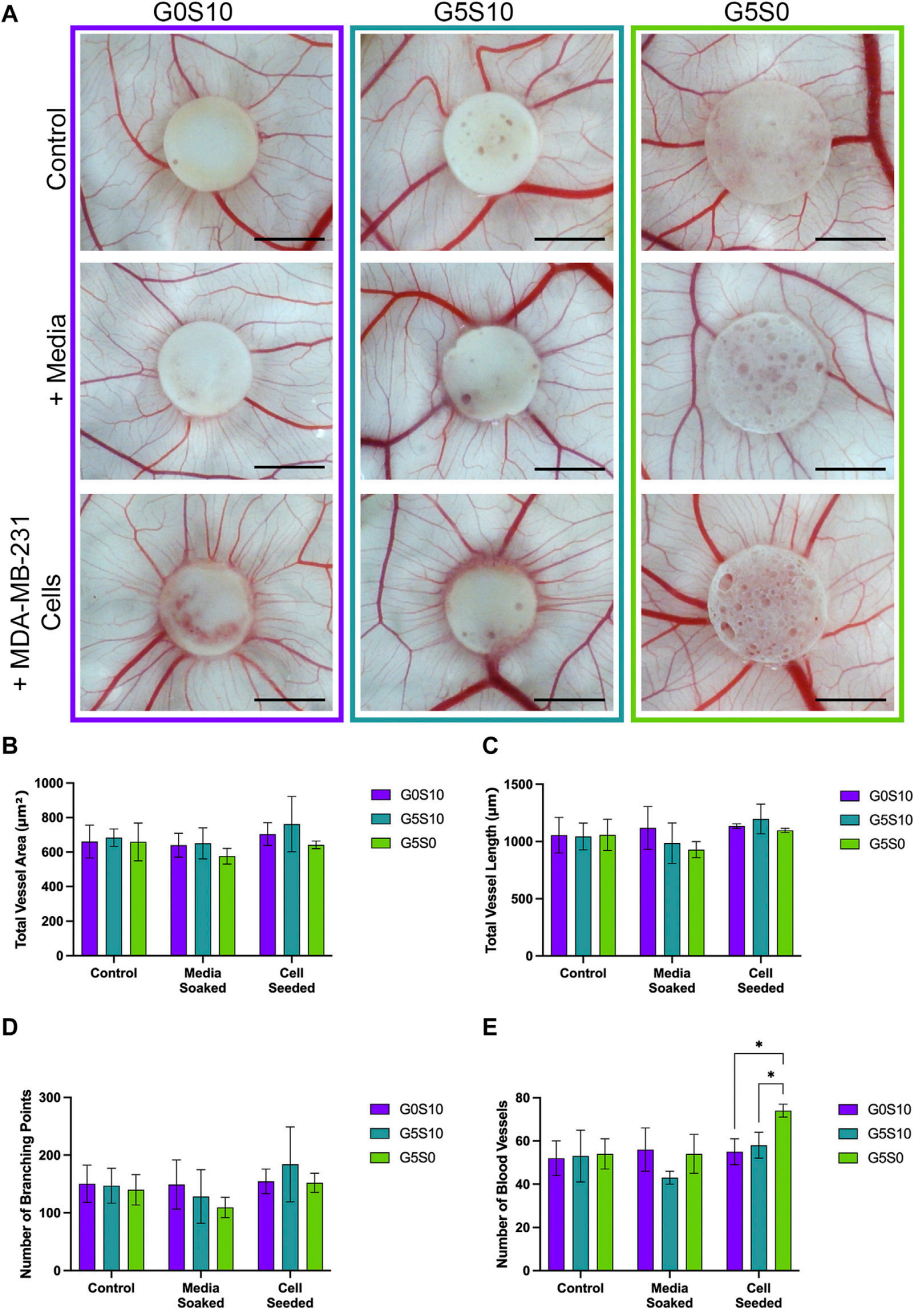
Assessment of the angiogenic potential of PCL-M polyHIPEs. **(A)** Digital Images of surfactant-only (G0S10), surfactant and gelatin-containing (G5S10) and gelatin-only (G5S0) PCL-M polyHIPEs prepared in control (PBS soaked), media soaked and cell seeded conditions on chick chorioallantoic membrane (CAM) at day 13 (scalebar represents 5 mm) with quantitative assessment describing **(B)** the total vessel area, **(C)** the total vessel length, **(D)** the number of branching points and **(E)** the number of blood vessels surrounding the PCL-M polyHIPE scaffolds (Mean ±SD, *N* = 3).

The *ex ovo* CAM assay was utilised to assess the angiogenic potential of PCL-M polyHIPEs seeded with MDA-MB-231 breast cancer cells. Visually, we observed no negative impact of the gelatin-containing scaffolds ± cancer cells on the viability of the chick embryos or vascular network (Figure 3A). We observed increased directionality of the vessels towards the scaffold when seeded with MDA-MB-231 cells. To further investigate this finding, we quantified the vessels, defining the total vessel area and length, number of branching points and the number of vessels surrounding the scaffolds. There was no significant difference in the total vessel area and length or number of branching points between the conditions (Figures 3B–D). There was a significant increase in the number of vessels surrounding the scaffold gelatin-only cell-seeded polyHIPE compared to the surfactant-containing cell-seeded polyHIPEs (Figure 3E).

**FIGURE 4:**
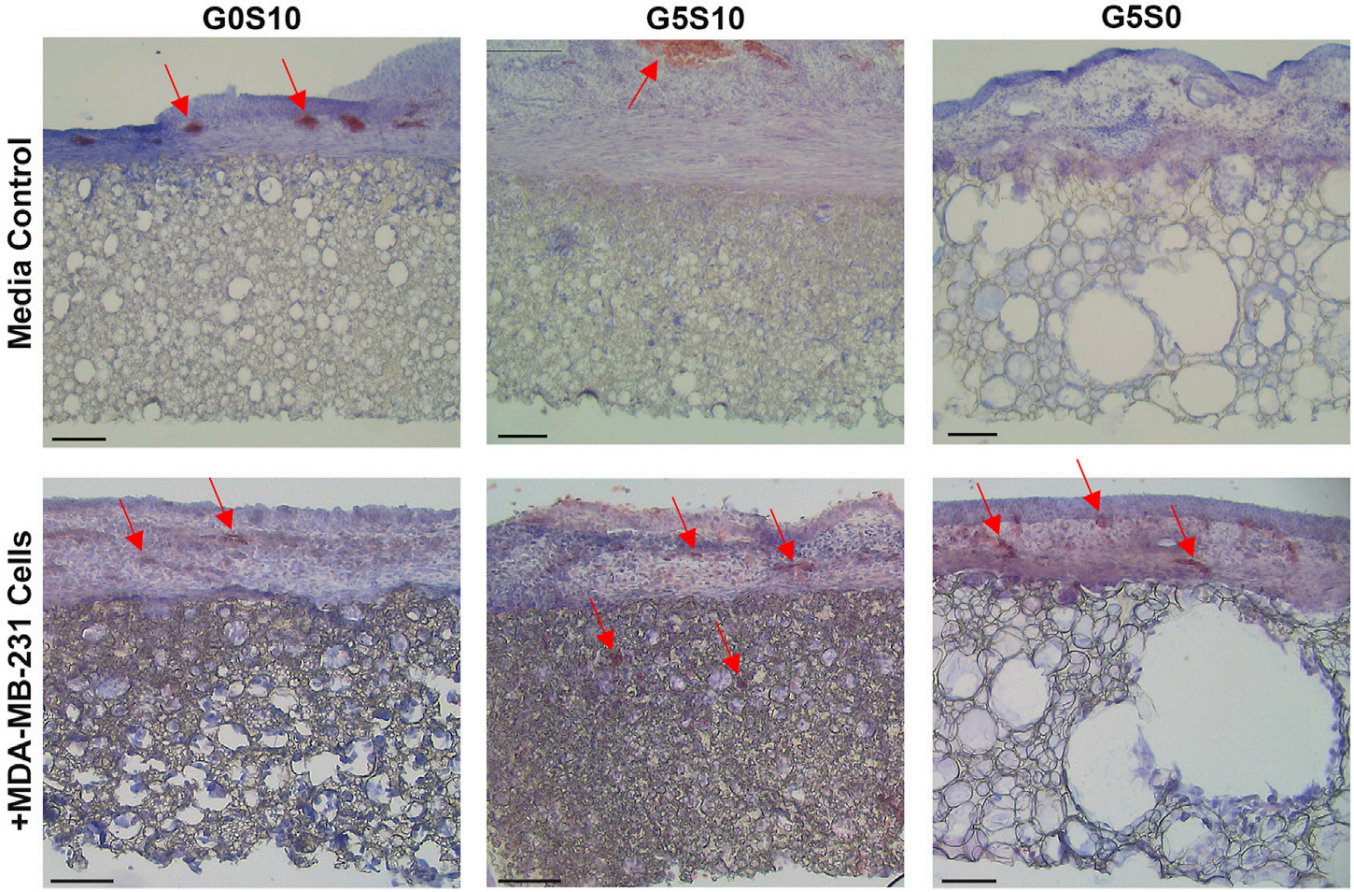
Haematoxylin and Eosin staining to analyse the angiogenic potential of surfactant-only (G0S10), surfactant and gelatin-containing (G5S10) and gelatin-only (G5S0) PCL-M polyHIPEs prepared in media soaked and cell-seeded conditions on chick chorioallantoic membrane (CAM) at day 13 (scalebar represents 100 μm, red arrows indicate blood vessels).

Haematoxylin and Eosin (H&E) staining was used to further assess the integration of the CAM vasculature within the gelatin-containing PCL-M polyHIPEs (Figure 4). Blood vessels (indicated by red arrows) were observed in the CAM tissue on the polyHIPE scaffolds, with more vessels present in the CAM tissue on the cell-seeded gelatin-containing scaffolds. Moreover, additional blood vessels were observed within the cell-seeded surfactant and gelatin-containing polyHIPE scaffolds.

## 5 Discussion

Within this study, we demonstrate the use of gelatin-containing PCL-M polyHIPEs that can support 3D breast cancer cell culture, whilst maintaining the key functionality of expressing vascular endothelial growth factor (VEGF) to promote angiogenesis. We used an *ex ovo* CAM assay to validate the capability of these gelatin-containing cell-seeded polyHIPEs for vascular ingrowth. Our study presents a potential substrate, with tuneable mechanical properties for use within microphysiological systems (MPS) that can successfully support 3D breast cancer cell culture and vascular ingrowth.

Firstly, we assessed the stiffness of the polyHIPE scaffolds to better understand the mechanical environment around the cells cultured within the substrates. We assessed the stiffness of the polyHIPEs in wet conditions with gelatin remaining in the scaffold to be more physiologically relevant, as has been discussed in previous studies (Aldemir Dikici et al., 2019; Jackson et al., 2023). Interestingly, when compared to our previous study in which PCL-M polyHIPE constructs were compressed in dry conditions with the gelatin removed prior to testing, we observe little difference in the stiffness of the resulting polyHIPEs (Figure 1A). Therefore, this suggests that the fabrication technique, utilising gelatin in the internal phase and the resulting effect it has on the polyHIPE structure is responsible for the change in stiffness rather than the condition of the scaffolds during testing (wet/dry ± gelatin).

When further analysing the stress-strain curves, all the polyHIPE samples demonstrated typical linear elastic behaviour at low strain with a final region of rapidly increasing stress at high strain, this is likely due to material densifying (Sun et al., 2016). The standard surfactant-only PCL-M polyHIPE displayed standard viscoelastic behaviour, as observed in our previous study (Furmidge et al., 2023). However, the addition of gelatin into the scaffolds alters the response of the polyHIPE to compressive loads, observing a deviation in the curve at 60% strain before increasing to a maximum (Figure 1B). The S-shaped curve observed, indicates elastic instability. Up to 60% strain we observed the stiffness due to the composition of the pores and the gelatin which results in a higher stiffness than the surfactant PCL-M polyHIPE (G0S10). 60%–75% strain is where we observed elastic instability, this is likely due to the gelatin being extruded from scaffold through the collapsed pores. We observed this difference visually, finding that the gelatin-containing polyHIPEs structurally failed following compression, with large sections of gelatin protruding from the broken body of the scaffold compared to surfactant-only polyHIPEs, which remained intact (Supplementary Figure S3). Moreover, gelatin has its own viscoelastic properties which differ from PCL-M, and as such, this may introduce a difference in energy dissipation and the resulting deformation (Guarino et al., 2017; Moučka et al., 2023). At 75% strain onwards the stiffness observed is due to the bulk of the PCL-M in which the gelatin-containing polyHIPE scaffolds demonstrate a similar stiffness to surfactant-only PCL-M polyHIPE scaffolds. In our previous study, compression of gelatin polyHIPE constructs in which the gelatin was removed prior to compression testing did not result in the S-shaped curve observed in this study (Furmidge et al., 2023). This would further suggest it is the gelatin remaining in the scaffold that causes the complex response to the compression rather than any physical changes to the structure of the polyHIPE when using a gelatin solution as the internal phase.

To ensure the presence of gelatin within the scaffolds following the post-processing washing steps and prior to cell seeding we used ATR-FTIR to identify the attributed peaks (Figure 2A). In both samples containing gelatin, amide I peaks were evident and interestingly, sample G5S0 shows the highest absorption in this region, which ties well with the fact that this sample contains gelatin only. Gelatin is widely used in biomaterials due to its cell adhesive properties and capability to provide a more ECM-like environment for cell growth (Bello et al., 2020; Lukin et al., 2022; Asim et al., 2023). Interestingly, the presence of gelatin did not have any significant effect on the metabolic activity of the cells compared to the surfactant-only polyHIPE (Figure 2B), these finding are similar to previous studies in which common coating techniques, such as fibronectin and plasma coating did not yield any significant improvement in 3D cell culture within PCL-M polyHIPEs (Dikici et al., 2019; Jackson et al., 2023). In this study the potential improvement in cell attachment might be mitigated by the lower amount of surface area or the potential for the cells to fall through the pores when being seeded in the larger pore scaffolds. However, this technique, which utilises a cost-effective, biocompatible protein, provides a simple and effective method to alter pore size and stiffness whilst supporting 3D cell culture. Thus, these scaffolds could be used in combination with each other within an MPS cancer model to achieve a diverse range of mechanical cues and environments to influence different stages of the metastatic cascade.

Vascular endothelial growth factor (VEGF) is a factor secreted by tumour cells, including the MDA-MB-231 breast cancer cell line used in this study. Expression of VEGF is a key factor for angiogenesis, promoting the proliferation of vascular endothelial cells. Using a VEGF ELISA kit, we confirmed the expression of VEGF from MDA-MB-231 cells cultured on surfactant-only and gelatin-containing PCL-M polyHIPEs (Figure 2C). There is variation within these results, and it is most likely due to the inefficiency of cell adhesion which arises from using a manual seeding technique. Further improvements in the seeding technique using automation would be beneficial in the future to reduce such variability. The concentration of VEGF expressed is comparable with previous studies which have used similar cell numbers (Matsui et al., 2008; Sohn et al., 2018). This further suggests that PCL-M polyHIPEs (surfactant-only and gelatin-containing) are suitable substrates for 3D cell culture of breast cancer.

It is well documented in the literature that gelatin is a suitable biomaterial to replicate the mechanical properties of breast tissue and has been commonly used to fabricate breast tissue phantoms (McGarry et al., 2020; Cannatà et al., 2021; Amiri et al., 2022). Therefore, whilst the stiffness of the PCL-M scaffolds does not replicate that seen in soft tissue *in vivo,* the gelatin within the structure can provide a more mimetic environment for the breast cancer cells. Furthermore, it has been widely reported that gelatin sponges implanted on CAM assays demonstrate good levels of angiogenesis (Ribatti et al., 1997; Ribatti et al., 2006; Dreesmann et al., 2007). The gelatin sponges used in many previous studies provide a permissive substrate for cell attachment, migration and proliferation resulting in vastly improved rates of angiogenesis compared to commercial collagen sponges. However, it is often difficult to handle and manipulate hydrogels. The combination of gelatin within a PCL-M polyHIPE scaffold provides a synthetic polymer scaffold, which is easily handled and manipulated, and a hydrogel to promote angiogenesis and vascular invasion within the bulk of the scaffold. Similarly, Tan et al. used PCL/gelatin electrospun scaffolds combined with induced pluripotent stem-cell derived endothelial cells (iPSC-ECs) (Tan et al., 2018). When implanted *in vivo* it was observed the iPSC-ECs survived a further 3 days once implanted and there was improved blood perfusion and host-angiogenic responses compared to when the iPSC-ECs were implanted without the composite PCL/gelatin scaffold.

In this study we seeded MDA-MB-231 cells on to gelatin-containing PCL-M polyHIPEs to further support angiogenesis by the expression of VEGF from the triple negative breast cancer cell line. Wang et al. have shown how the addition of VEGF to a PCL/gelatin electrospun scaffold can improve endothelial cell proliferation *in vitro* and enhanced vascularisation *in vivo* (Wang et al., 2015). Sustained release of VEGF was achieved by functionalising the gelatin by heparin immobilisation, creating a binding site for VEGF. Similarly, Del Gaudio et al. functionalised gelatin by crosslinking with genipin, resulting in improved angiogenesis (Del Gaudio et al., 2013). Alternatively, Jiang et al. combined PCL nanofibers with gelatin encapsulated VEGF to enhance angiogenesis of endothelial cells (Jiang et al., 2018). In this study, we simplify the scaffold processing steps, removing the need for gelatin functionalisation or encapsulation by utilising the innate ability of MDA-MB-231 cells to express VEGF.

To validate the use of gelatin-containing PCL-M polyHIPEs to support vascular invasion and growth, we used the *ex ovo* CAM assay, assessing the effect of cell-seeded PCL-M polyHIPEs on angiogenesis from an established, pre-existing vascular network. The CAM assay has been well documented to study the angiogenic capability of biomaterial scaffolds (Naik et al., 2018; Ribatti et al., 2020). Due to the biological nature of the CAM assay, it is common to see variation within datasets as shown in previous studies which utilise the CAM assay (Aldemir Dikici et al., 2020; Samourides et al., 2020). We report visually increased directionality of the vessels towards the cell-seeded polyHIPE scaffolds (Figure 3). We suggest this observation is due to the expression of VEGF from the breast cancer cells promoting directed vessel growth around the circumference of the scaffolds. This is further supported in literature, in which VEGF is identified as one of the main factors for vascular growth regulation in the CAM (Chen et al., 2021), with many studies observing an increase in scaffold integration and number of blood vessels when the scaffolds were loaded with VEGF (Cidonio et al., 2019a; Cidonio et al., 2019b; He et al., 2019; Marshall et al., 2020). Moreover, similar directional vessel growth has been observed by Guerra et al. in response to varying VEGF concentrations (Guerra et al., 2021). They identified that increased levels of VEGF resulted in vessel growth with increased vessel density towards the VEGF-loaded hydrogel.

There was a statistically significant increase in the number of vessels surrounding the cell-seeded gelatin-only polyHIPE scaffolds (Figure 3E). This was also observed in the H&E staining, we observed there were more vessels present in the CAM tissue on the cell-seeded gelatin-only polyHIPE scaffold compared to the media-soaked gelatin-only polyHIPE scaffold (Figure 4). A study investigating porous poly(glycerol sebacate urethane) scaffolds observed a significant increase in the number of vessels surrounding scaffolds which had larger pores (Samourides et al., 2020). This correlates to the significance observed in this study in which the gelatin-only scaffolds have significantly larger pores than the surfactant-containing polyHIPEs. The average pore sizes have been previously reported, as 80 µm for the gelatin-only scaffold, 39 µm for the gelatin and surfactant scaffold, and 53 µm for the surfactant-only scaffold (Furmidge et al., 2023). On the other hand, whilst we observed a greater number of vessels in the CAM tissue around the gelatin-only scaffolds, we did not observe vessels within the scaffold. The H&E staining of these scaffolds showed the CAM tissue forming a distinct layer on the scaffold with limited integration. This lack of integration was further pronounced when preparing the samples for histological assessment, and the layer of CAM tissue was easily separated from the scaffold, as previously described by Mangir et al. (2019). This lack of integration is likely due to the larger pores in the gelatin-only PCL-M polyHIPEs. Whilst large pores are favourable for vascular invasion and integration, for cell attachment and migration they may need to be significantly smaller. The ingrowing CAM cells are likely fibroblasts, and it is reported that fibroblast ingrowth occurs with scaffold pore sizes of 5–15 µm (Yang et al., 2001). Interestingly, on multiple occasions in the cell-seeded surfactant and gelatin-containing PCL-M polyHIPEs, we observed the CAM membrane starting to grow over and envelop the scaffold (Figure 3. G5S10 +MDA-MB-231; Supplementary Figure S4). This phenomenon has been observed previously as an indicator of tissue/scaffold integration and angiogenesis (Baiguera et al., 2012; Totonelli et al., 2012; Orlando et al., 2013). Interestingly, these studies use ECM-derived matrices to fabricate decellularised scaffolds. Thus, it is likely the gelatin, a heterogenous mixture of peptides which are derived from Collagen, which is one of the most abundant ECM proteins, alongside the expression of VEGF from the MDA-MB-231 cells within the PCL-M polyHIPEs is responsible for stimulating the envelopment of these scaffolds. The H&E staining provided further evidence of vascular integration, where we observed the presence of blood vessels within the surfactant and gelatin-containing scaffold (Figure 4). Whilst these scaffolds have smaller pores [mean = 39 µm (Furmidge et al., 2023)], vascular invasion was observed. Similarly, Paterson et al. demonstrated vascular invasion occurred within emulsion-templated microspheres with small pores (median pore size = 21 µm) however, only in combination with human embryonic stem cell-derived mesenchymal progenitor cells (Paterson et al., 2018). Furthermore, they suggest the expression of VEGF from the cells may be partially responsible for the induction of the angiogenic response. Furthermore, Baker et al. observed vascular ingrowth within porous PCL scaffolds with a similar range of pore sizes as the scaffolds used in this study (Baker et al., 2011). The study also identified that the infiltrating vascular network preferentially aligned along micro-fractures in the structure. Therefore, any similar fractures or weaknesses within the polyHIPE structure could provide additional support for vascular alignment and infiltration and is a possible design feature to investigate in the future to further improve the vascularisation of gelatin-containing PCL polyHIPEs.

## 6 Conclusion

In this study, we demonstrated the use of gelatin-containing PCL-M polyHIPEs to support 3D breast cancer cell culture by assessing the cell metabolic activity and the expression of VEGF. Furthermore, we validated the use of these substrates to support vascular invasion and growth using the CAM assay. Via combining breast cancer cells with the gelatin-containing polyHIPE substrates, we observed a significant increase in the number of blood vessels surrounding the scaffold and improved tissue integration. Thus, we present gelatin-containing PCL-M polyHIPEs as a promising composite material for MPS substrates to support both 3D cancer cell culture and vascular ingrowth.

## Data Availability

The raw data supporting the conclusions of this article will be made available by the authors, without undue reservation.
