# Supplementary material for: Gelatin-containing porous polycaprolactone PolyHIPEs as substrates for 3D breast cancer cell culture and vascular infiltration
Source: Front Bioeng Biotechnol. 2024 Jan 8;11:1321197. doi: 10.3389/fbioe.2023.1321197 (PMC10800367; doi:10.3389/fbioe.2023.1321197)
Supplement: Supplementary file 1 [file DataSheet1.docx]

***Supplementary Material***

**Gelatin-containing Porous Polycaprolactone PolyHIPEs as Substrates for 3D Breast Cancer Cell Culture and Vascular Infiltration**

**Caitlin E. Jackson^1,2^, Iona Doyle^1^, Hamood Khan^1^, Samuel F. Williams^3^, Betul Aldemir Dikici^4^, Edgar Barajas Ledesma^1^, Helen E. Bryant^5^, William R. English^6^, Nicola H. Green^1,2^, Frederik Claeyssens^1,2*^**

*** Correspondence:** f.claeyssens@sheffield.ac.uk

# Supplementary Figures


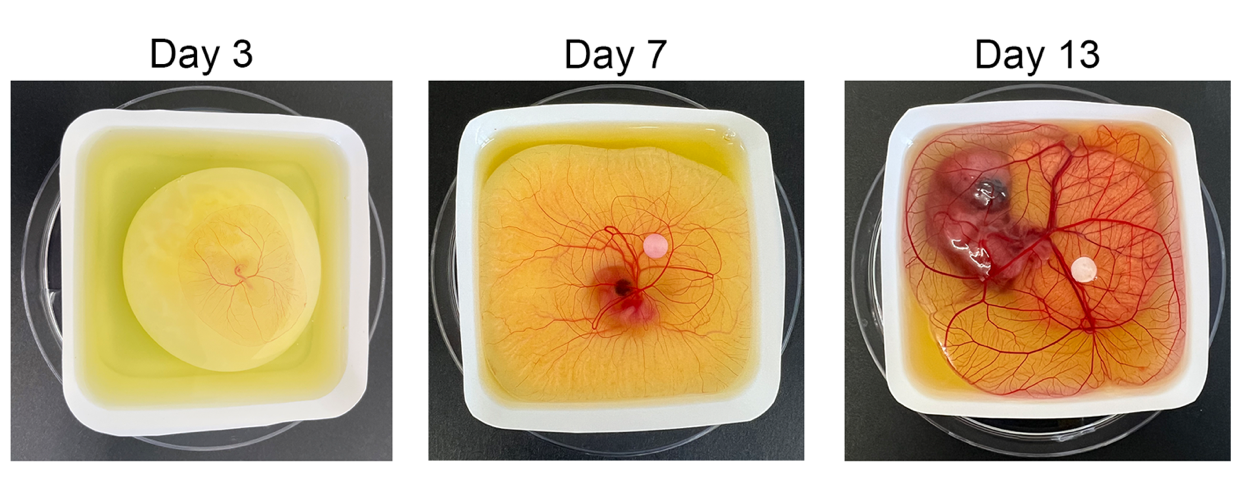


**SUPPLEMENTARY FIGURE 1.** The growth of the chick foetus and chorioallantoic membrane from cracking (day 3), to scaffold placement (day 7), to imaging (day 13).


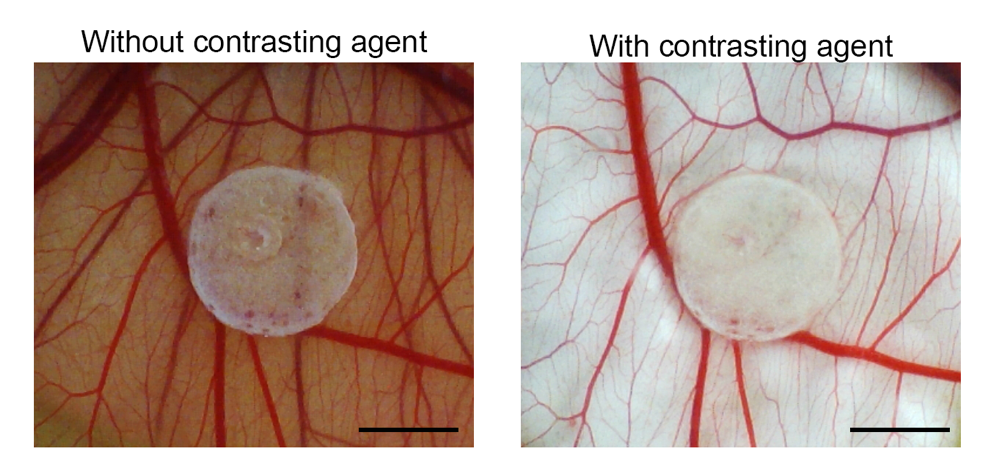


**SUPPLEMENTARY FIGURE 2.** Digital images demonstrating the effect of using a contrasting agent to improve the clarity of the vessels lying directly under the PCL-M polyHIPE scaffolds.


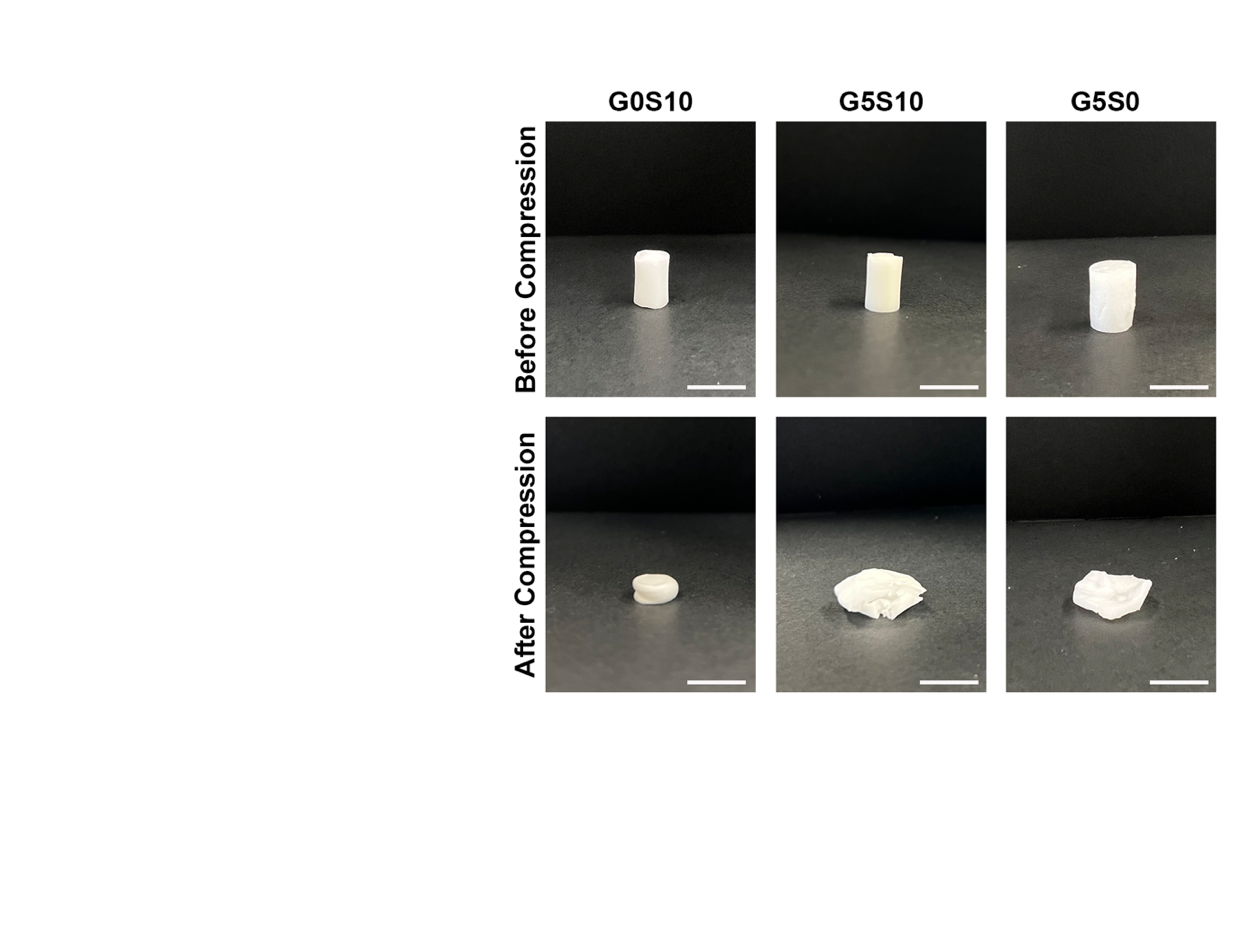


**SUPPLEMENTARY FIGURE 3.** Digital images, demonstrating the physical deformation experienced by PCL-M polyHIPEs containing gelatin following mechanical compression.


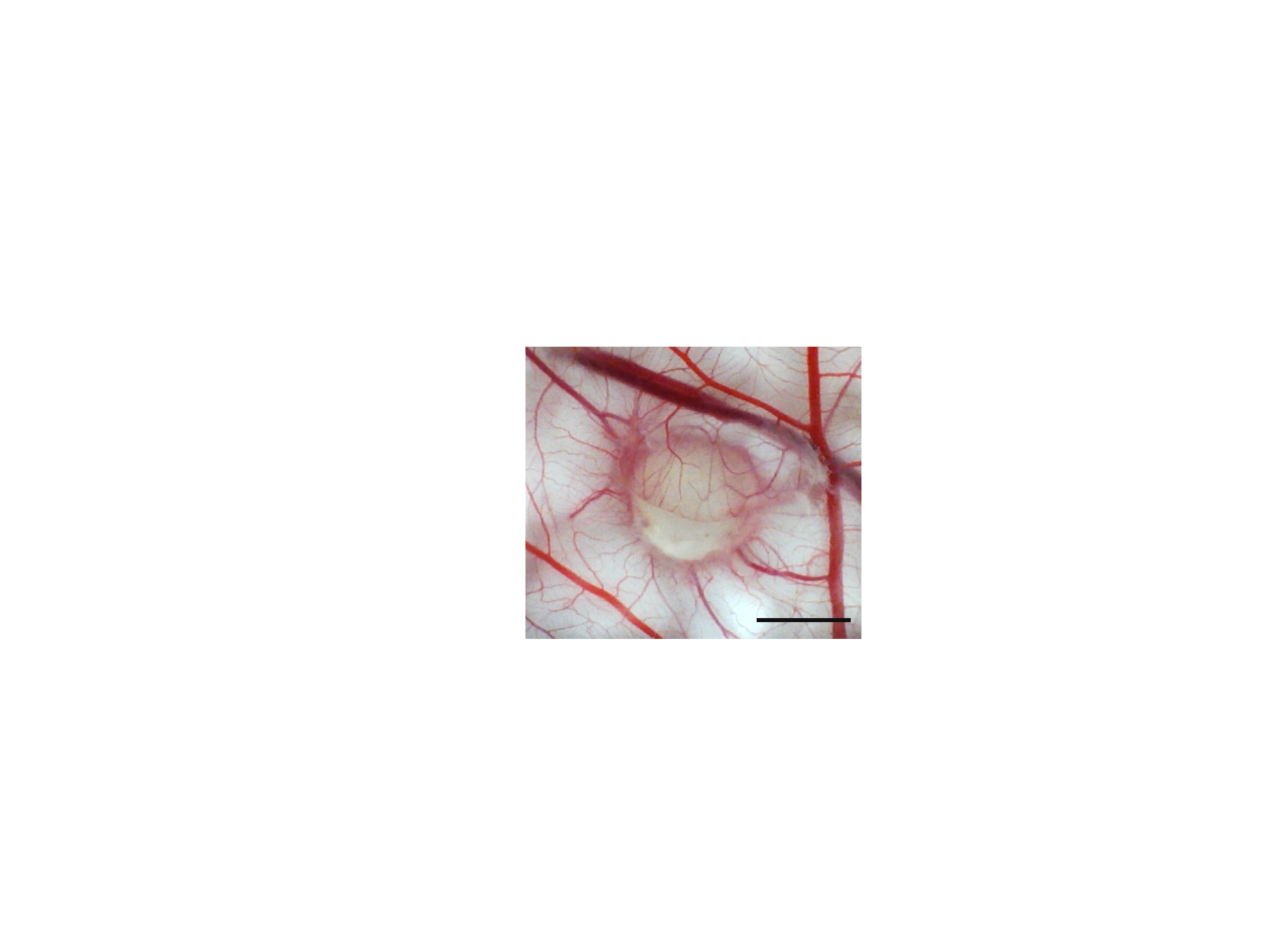


**SUPPLEMENTARY FIGURE 4.** Surfactant and gelatin-containing PCL-M polyHIPE enveloped by the chorioallantoic membrane (CAM).
